# Supplementary material for: Photoreceptors generate neuronal diversity in their target field through a Hedgehog morphogen gradient in Drosophila
Source: eLife. 2022 Aug 25;11:e78093. doi: 10.7554/eLife.78093 (PMC9507128; doi:10.7554/eLife.78093)
Supplement: Supplementary file 1. [file elife-78093-supp1.docx]

**Supplementary File 1: Table showing summary statistics of a mixed effects linear model for *ptc-lacZ* *(β-Gal)*** **MFI as a function of Distance and Cell position (distal-proximal).**

| **Source** | **F Ratio** | **Prob > F** |
| --- | --- | --- |
| Distance (μm) | 7744.904 | <.0001 |
| Cell position | 2320.251 | <.0001 |
| (Distance)*(Cell position) | 191.2012 | <.0001 |
