# Supplementary material for: Photoreceptors generate neuronal diversity in their target field through a Hedgehog morphogen gradient in Drosophila
Source: eLife. 2022 Aug 25;11:e78093. doi: 10.7554/eLife.78093 (PMC9507128; doi:10.7554/eLife.78093)
Supplement: Supplementary file 2. — (Note that only female genotypes are listed though both sexes were included in our analyses). [file elife-78093-supp2.docx]

**Supplementary File 2: Table listing all genotypes and experimental conditions used by figure panel.** (Note that only female genotypes are listed though both sexes were included in our analyses)

| **Fig.** | **Panel** | **Genotype** | **Conditions** |
| --- | --- | --- | --- |
| 1 | B | *Canton S* | Raised at 25^o^C |
| 1 | C | *Canton S* | Raised at 25^o^C |
| 1 | I | *;;ptc-lacZ* | Raised at 25^o^C |
| 2 | A, B | *;hh-sfGFP/+;* | Raised at 25^o^C |
| 3 | A, B, D | *y,w,hsflp^122^/+, Tub-Gal4, UAS-nls.GFP; FRT42D, Tub-Gal80 /FRT42D, ptc^S2^;* | Raised at 25^o^C before and after heat shocking. See Mosaic analysis for more detail. |
| 3 | C | *Canton S* | Raised at 25^o^C |
| 3 | E | *ey-Gal80; Gal80ts; R27G05-Gal4/UAS-CD8::GFP* | Raised at 18^o^C till late-L1, then shifted to 29^o^C for 72 hours |
| 3 | F | *ey-Gal80; Gal80ts; R27G05-Gal4/UAS-Ci^rep^* | Raised at 18^o^C for 12 days and 21 hours, then shifted to 29^o^C for 12 hours |
| 3 | G | *ey-Gal80; Gal80ts; R27G05-Gal4/UAS-Ci^RNAi^* | Raised at 18^o^C for 12 days, then shifted to 29^o^C for 24 hours |
|  |  |  |  |
| 4 | A-C | *Canton S* | Raised at 25^o^C |
| 4 | D-F | *ey-Gal80; Gal80ts; R27G05-Gal4/UAS-Ci^rep^* | Raised at 29^o^C |
| 4 | G-I | *ey-Gal80; Gal80ts; R27G05-Gal4/UAS-Ci^rep^* | Raised at 25^o^C |
| 5 | A | *y,w;;hh^ts2^/+ (24hrs ts)* | Raised at 18^o^C for 13 days, then shifted to 29^o^C for 24 hours |
| 5 | B | *y,w;;hh^ts2^* | Raised at 18^o^C for 14 days |
| 5 | C | *y,w;;hh^ts2^ (6h ts)* | Raised at 18^o^C for 13 days, then shifted to 29^o^C for 6 hours |
| 5 | D | *y,w;;hh^ts2^ (12h ts)* | Raised at 18^o^C for 12 days and 21 hours, then shifted to 29^o^C for 12 hours |
| 5 | E | *y,w;;hh^ts2^ (24h ts)* | Raised at 18^o^C for 12 days, then shifted to 29^o^C for 24 hours |
| 5 | F | *y,w;;hh^ts2^ (45h ts)* | Raised at 18^o^C for 11 days, then shifted to 29^o^C for 45 hours |
| 5 | G | *y,w;;hh^ts2^ (72h ts)* | Raised at 18^o^C for 10 days, then shifted to 29^o^C for 72 hours |
| 6 | A | *;;ry^503^, hh^P30^* | Raised at 25^o^C |
| 6 | B | *w*; GMR>+* | Raised at 29^o^C |
| 6 | C | *w*; GMR>hh^RNAi^* | Raised at 29^o^C |
|  |  |  |  |
| 1-fig. supp. 1 | G-K | *Canton S* | Raised at 25^o^C |
| 3-fig. supp. 1 | A | *y,w,hsflp^122^/+, Tub-Gal4, UAS-nls.GFP; FRT42D, Tub-Gal80 /FRT40A, smo^3^;* | Raised at 25^o^C before and after heat shocking. See Mosaic analysis for more detail. |
| 3-fig. supp. 1 | B | *ey-Gal80; Gal80ts; R27G05-Gal4/UAS-CD8::GFP* | Raised at 18^o^C till late-L1, then shifted to 29^o^C for 72 hours |
| 4-fig. supp. 1 | A | Same as 3E | Same as 3E |
| 4-fig. supp. 1 | B | Same as 3F | Same as 3F |
| 4-fig. supp. 1 | C | *ey-Gal80; Gal80ts; R27G05-Gal4/UAS-Ci^rep^* | Shifted to 25^o^C |
| 5-fig. supp. 1 | A-E, F- J | Same as 5A-G | Same as 5A-G |
